# Developmental differences in brain functional connectivity during social interaction in middle childhood

**DOI:** 10.1016/j.dcn.2022.101079

**Published:** 2022-01-31

**Authors:** Yaqiong Xiao, Diana Alkire, Dustin Moraczewski, Elizabeth Redcay

**Affiliations:** a Center for Language and Brain, Shenzhen Institute of Neuroscience, Shenzhen, China; b Department of Psychology, University of Maryland, College Park, MD, USA; c Neuroscience and Cognitive Science Program, University of Maryland, College Park, MD, USA; d Data Science and Sharing Team, National Institute of Mental Health, Bethesda, MD, USA

**Keywords:** Mentalizing, Social reward, FMRI, Functional connectivity, Middle childhood

## Abstract

The transition from childhood to adolescence is marked by significant changes in peer interactions. However, limited research has examined the brain systems (e.g., mentalizing and reward networks) involved in direct peer interaction, particularly during childhood and early adolescence. Here, we analyzed fMRI data from 50 children aged 8–12 years while they participated in a task in which they chatted with a peer (Peer) or answered questions about a story character (Character). Using a beta-series correlation analysis, we investigated how social interaction modulates functional connectivity within and between mentalizing and reward networks and whether this modulation changes with age. We observed effects of social interaction on functional connectivity were modulated by age within the mentalizing and reward networks. Further, greater connectivity within and between these networks during social interaction was related to faster reaction time to the Peer versus Character condition. Similar effects were found in the salience and mirror neuron networks. These findings provide insights into age-related differences in how the brain supports social interaction, and thus have the potential to advance our understanding of core social difficulties in social-communicative disorders, such as autism spectrum disorder.

## Highlights

• We examined brain functional connectivity in a social-interactive context.
• The effect of social partner on mentalizing and reward networks was modulated by age.
• Connectivity within and between these networks is related to behavioral performance.
• These effects are also found in salience and mirror neuron networks.

## 1 Introduction

Social interaction is essential for our everyday lives. From the first moments after birth, we interact with others; throughout the lifespan, we seek out partners, develop social relationships, and importantly, learn from social interactions. Theoretical work suggests that both social-cognitive and social-motivational networks may play important and integrated roles in social interaction (for reviews, see Chevallier et al., 2012; Krach et al., 2010; Ruff and Fehr, 2014) and that these social-affective and cognitive networks undergo functional changes both within and between networks during key phases of social development, such as the transition to adolescence when peers become more salient (Nelson et al., 2016). However, while recent work has highlighted the contributions of these two networks to social interaction (for a review, see Redcay and Warnell, 2018), very little work has tested how functional connectivity within and between these networks may contribute to social interaction. Further, no study has examined age-related differences in network organization in social-interactive contexts during this transition period (i.e., from childhood to adolescence).

Successful social interaction requires mentalizing, or theory of mind, the ability to infer one’s own or others’ mental states (Schurz et al., 2014). A group of regions, referred to together as the “mentalizing network,” has been identified based on third-person (i.e., non-interactive) explicit tasks (e.g., tasks in which participants reason about a story character’s beliefs) that contrast mental with non-mental reasoning. These regions include bilateral temporal-parietal junction (TPJ), bilateral anterior temporal lobes (ATL), precuneus (PC), ventromedial prefrontal cortex (vmPFC), and dorsomedial prefrontal cortex (dmPFC) (Molenberghs et al., 2016, Schurz et al., 2014). Developmentally, these regions show increasing selectivity and greater functional network integration that is related to both age and social-cognitive abilities (Gweon et al., 2012, Richardson et al., 2018).

In addition to the mentalizing network, theoretical (Rekers et al., 2011, Tomasello et al., 2005, Tomasello and Carpenter, 2007) and empirical work (Chevallier et al., 2012, Hill, 1987, Krach et al., 2010, Krach et al., 2008, Leary, 2010, Rilling and Sanfey, 2011, Tamir and Mitchell, 2012) suggest a key role of motivation and reward processes in social interaction. These processes are supported by a reward network, including the orbitofrontal cortex (OFC), vmPFC, anterior cingulate cortex (ACC), bilateral ventral striatum (VS), and bilateral amygdala (Knutson et al., 2001, McClure et al., 2004; for a review, see Haber and Knutson, 2010). Theoretical work suggests that these reward-relevant regions may communicate with regions of the mentalizing network described above in socially-motivating contexts (Ruff and Fehr, 2014). However, gaps remain in our understanding of how these networks work together during social interaction due to two main limitations of prior work.

A first limitation is that most of our understanding of the mentalizing and reward networks comes from non-interactive studies. However, “second-person” neuroscience studies (i.e., those in which the participant is engaged in interaction) demonstrate that the brain likely responds to social stimuli differently in interactive compared to non-interactive contexts (Redcay and Schilbach, 2019, Redcay and Warnell, 2018, Schilbach et al., 2013). For example, in interactive studies, mentalizing regions are engaged spontaneously during social interaction even in the absence of explicit mentalizing demands (Alkire et al., 2018, Redcay and Schilbach, 2019, Rice et al., 2016, Rice and Redcay, 2016, Warnell et al., 2018). Further, reciprocal engagement with a real-time social partner elicits greater activation within these reward sensitive regions than non-interactive social processing (Pfeiffer et al., 2014, Schilbach et al., 2010, Redcay et al., 2010, Alkire et al., 2018). Further, children responded more quickly and reported higher subjective reward when interacting with a social partner (i.e., interactive) compared to answering questions about a story character (i.e., non-interactive) (Alkire et al., 2018). Thus, fully understanding the developmental neural correlates of social interaction requires examining social interaction within an interactive context.

A second limitation is that much of the work described above has characterized regions that are sensitive to various aspects of social interaction by measuring their activation, or average stimulus-evoked response across repeated trials. In contrast, functional connectivity, or the temporal correlation of activity between regions provides information on how regions or networks communicate with each other during different task contexts. Characterizing brain function in this way—that is, beyond the activation of individual regions—allows examining novel questions that cannot be addressed with activation analyses alone. For example, while language and mentalizing regions show functionally distinct activation profiles, they demonstrate significant functional connectivity during language comprehension, suggesting distinct but integrated roles of these networks during language comprehension (Paunov et al., 2019). In the context of social interaction, theoretical work suggests that engaging in real-time social interaction (compared to non-interactive contexts) may lead to greater functional connectivity (or integration) among networks relevant to social processing (Redcay and Schilbach, 2019, Schilbach et al., 2013). However, while research has demonstrated coactivation of regions within reward and mentalizing networks during social reward processing (Izuma et al., 2008, Powers et al., 2013; for a review, see Ruff and Fehr, 2014), direct evidence of functional connectivity between regions in the reward and social-cognitive networks during social interaction is still lacking, leaving gaps in our understanding of whether and how reward and social-cognitive networks function together.

This prediction that social interaction will drive greater connectivity between regions within mentalizing and reward networks is based on the growing second-person neuroscience research in adults. However, this question has not been addressed developmentally. Middle childhood, a period of transition to early adolescence, contains significant changes in social interaction and social relationships, including greater time spent with and more attention to peers (Devine and Hughes, 2013, Farmer et al., 2015, Parker et al., 2015, Rubin et al., 2007). The social re-orientation theory posits that the enhanced sensitivity to peers during early adolescence is associated with changes in brain networks involved in social functions (Nelson et al., 2016). During this period of transition to adolescence (i.e., middle childhood) peers may become more motivating and this may lead to greater integration between motivational and social-cognitive networks when engaged with a peer compared to another social stimulus.

In the present study, we examined functional connectivity of mentalizing and reward networks during a social-interactive task in 8–12 year-olds using an interactive social prediction paradigm (Alkire et al., 2018). We predicted that reward and mentalizing networks would be correlated but that they would show greater within than between network connectivity across conditions, suggesting they are functionally distinct. We further predicted modulations in connectivity based on social context. Specifically, making predictions about a peer (i.e., real-time social partner) would result in greater connectivity within and between reward and mentalizing networks than making predictions about a story character (i.e., non-interactive). Finally, we predicted this connectivity would be modulated by age, with increasing integration between mentalizing and reward systems with age as peers become more salient and motivating. Behaviorally, as previous work has shown faster reaction time (RT) related to higher social motivation (Chevallier et al., 2016; DiMenichi and Tricomi, 2015), we predicted higher motivation (e.g., faster RT and higher subjective reward) when making predictions about a peer versus story character, and that this increases with age. Further, we predicted that children who demonstrate greater social motivation (i.e., faster RT and greater subjective reward to Peer versus Character condition) would show greater functional connectivity within and between reward and mentalizing networks.

## 2 Material and methods

### 2.1 Participants

We recruited 65 children using a database of families in the Washington, DC, metropolitan area to participate in behavioral and MRI sessions at the University of Maryland. Fifteen of them were excluded after either the behavioral or MRI visit for the following reasons: Five children had excessive motion in the scanner (see *fMRI data analysis* section below for detailed descriptions), six children did not believe the illusion of peer interaction, one child did not finish the scan due to a technical error, one child scored low in both IQ (< 85) and reading fluency (< the 3rd grade level) tests, and one child scored low in the reading fluency test (< the 3rd grade level). The final sample included 50 children (30 males, mean age 10.38 ± 1.33 years, range 8.18–12.97 years) with available behavioral measures (see *Behavioral assessments* section below) and MRI data. The age distribution of these participants is shown in Fig. S1A. Among 50 subjects, 10% identified as Hispanic/Latino, 60% as White/Caucasian, 22% as Black/African American, and 18% as more than one race, with race and ethnicity (Hispanic/Latino or not) information collected separately. Eighty-six percent of participants are from families reporting over $75,000 in total family income, 6% from families reporting family income between $35,000–$75,000%, and 4% from families reporting family income less than $35,000 (4% did not report on income). Prior to participating, all participants gave informed assent and their parents gave informed consent. They were full-term, native English speakers, without any MRI contraindication, diagnosis of neurological or psychiatric disorder, or first-degree relatives with autism or schizophrenia. All MRI and behavioral data were collected between 2017 and 2018. This study was approved by the University of Maryland Institutional Review Board. A subsample of this cohort has been reported in a prior study (Alkire et al., 2018).

### 2.2 Behavioral assessments

On a separate visit prior to the scan, each child completed a battery of behavioral measures including the Kaufman Brief Intelligence Test Second Edition (KBIT-2; Kaufman and Kaufman, 2004) and reading fluency (Woodcock and McGrew, 2001). Total IQ (standard score ≥ 85) and reading fluency (≥ 3rd grade level) served as screening criteria for participation in the current study; as aforementioned, two children were excluded from the final sample due to low scores in these two tests.

### 2.3 fMRI design and procedure

We used a 2 * 2 event-related design with factors of mentalizing (Mental and Non-Mental) and social interaction (Peer and Character) (Alkire et al., 2018). In the experiment, we told participants they would be chatting with an age- and gender-matched peer in another lab who would also be participating in an MRI study, when in reality the task consisted of pre-programmed responses. To enhance the peer illusion, we took a picture of the participants before the scan under the guise of exchanging it with another lab and asked them to choose a chat partner from two pictures of children matched on age and gender. The photos of children were selected from the NIMH Child Emotional Faces Pictures Set (Egger et al., 2011) as well as from Getty Images (www.gettyimages.com) and Google Images, with the goal of matching the racial and ethnic diversity of our participants. In addition, participants were aware that they would be chatting with and making predictions about the peer half of the time and “chatting” with the computer and making predictions about a fictional character of the same gender and age as the participant for the other half of the time.

In the scanner, participants were engaged in an interactive game and made predictions about either the peer or the character. Each trial consisted of two periods: Guess (8 s) and Feedback (2 s). The Guess period included receiving a hint and making a prediction based on that hint. Specifically, participants were presented with the name of the chat partner (Peer condition) or “Computer” (Character condition), followed by a one-sentence hint about the peer (e.g., “I think flying is fun”) or the story character (e.g., “Mia likes to run”), which either contained a mental state (Mental, e.g., beliefs, desires, preferences, emotions, and knowledge) or not (Non-Mental, e.g., facts or situations about the peer or character). Then, participants answered either “Which will I/she/he pick” (Mental) or “Which of these match” (Non-Mental) by choosing between two choices via pressing the button. In the Feedback period, participants learned whether their predictions matched the answers of the peer or the computer. The experimental design is shown in Fig. S2. This 2 * 2 design included four conditions: Peer Mental, Peer Non-Mental, Character Mental, and Character Non-Mental, with 24 trials for each condition and a total of 96 trials in the task. We determined the trial distribution and inter-stimulus/trial intervals using Design Explorer (Moraczewski et al., 2016 unpublished software) as it minimizes collinearity between events in the design matrix. Next, we submitted the resulting matrix to AFNI’s 1d_tool program (Cox, 1996) which confirmed minimal correlations between regressors of interest.

The presentation of the trials was performed by PsychoPy (Peirce, 2009) with 4 runs of 24 trials. A fixation cross was presented for 10 s at the beginning and 15 s at the end of each run, and the chat partner’s photo appeared at the end of each run. Trials were separated by a fixation cross presented for a jittered 2–6 s, centered around 3.5 s and distributed exponentially. The Guess and Feedback periods were separated by a fixation cross with the same jittered parameters.

### 2.4 In-scanner performance

We measured participants’ performance (i.e., accuracy and RT) during the fMRI task across the four conditions, in which RT was a proxy measure of social motivation. To examine whether behavioral performance was modulated by social interaction or mentalizing and whether the modulation of social interaction changed with age, we used mixed effects models to test the effects of social interaction, mentalizing, age, and the interaction effect of social interaction and age with subjects as a random effect, controlling for gender and IQ. For the main effect of social interaction, we performed a post-hoc paired *t*-test with mean RT or accuracy in Peer versus Character condition. For interaction effects of social interaction and age, we ran a post-hoc regression analysis controlling for gender and IQ.

### 2.5 Post-scan assessments

Following the scan, we asked participants to complete a post-scan questionnaire to assess their subjective experience on the interactive task. All ratings were made on a 5-point scale. These questions attempted to assess: (1) subjective reports of enjoyment when interacting with the peer and making predictions about the story character (i.e., Liked Chatting, Liked Guessing, Felt When Matched); (2) subjective reports of motivation for social-interactive (i.e., making predictions about the peer) and non-interactive (i.e., making predictions about the story character) conditions (i.e., Wanted to See); (3) subjective reports of attention for social-interactive (i.e., making predictions about the peer) and non-interactive (i.e., making predictions about the story character) conditions (i.e., Paid Attention); (4) subjective reports of difficulty when making predictions (Perceived Difficulty). Detailed questions for Peer and Character conditions are shown in Table S1.

We examined whether children’s subjective experience, including Liked Chatting, Liked Guessing, Felt When Matched, Wanted to See, Paid Attention, and Perceived Difficulty, was modulated by social context in the interactive task, i.e., Peer versus Character. Specifically, we tested the effects of social interaction, age, and their interaction, controlling for gender and IQ.

We tested whether children’s subjective reports of enjoyment (Liked Chatting, Liked Guessing, Felt When Matched), attention (Paid Attention), and motivation (Wanted to See) were correlated with RT as they are all measures of social motivation.

At the completion of the study, children and parents were debriefed about the deception. We used both the post-scan questionnaire and debriefing to probe participants’ belief in the peer illusion, and excluded six children from the analysis as they showed doubts or disbelief in the illusion.

### 2.6 Data acquisition

MRI data were collected at the Maryland Neuroimaging Center on a 3 T Siemens scanner (MAGNETOM Trio) with a 32-channel head coil. Four task runs were acquired using multiband-accelerated echo-planar imaging (66 interleaved axial slices, voxel size = 2.19 × 2.19 × 2.20 mm, TR/TE = 1250/39.4 ms, flip angle = 90, a multiband factor of 6), a total of 352 volumes and lasting 7 min 20 s per run. The following structural scan was obtained with a three-dimensional T1 magnetization-prepared rapid gradient-echo sequence (192 contiguous sagittal slices, voxel size = 0.45 × 0.45 × 0.90 mm, RT/TE = 1900/2.32 ms, flip angle = 9°). The first 4 volumes of each run were automatically discarded to allow for magnetization equilibrium.

### 2.7 fMRI data preprocessing

Neuroimaging data were preprocessed using fMRIPrep 1.4.1 (Esteban et al., 2019). Briefly, anatomical images were segmented and normalized to MNI space; functional images were skull-stripped, susceptibility distortion corrected, realigned, slice-time corrected, coregistered and warped to the normalized anatomical image (see https://osf.io/u62ef/ for full report of the preprocessing pipeline).

In order to account for the head motion, the voxel-wise framewise displacement (FD) was calculated following Power et al. (2012) to quantify head movements in the scanner and to control it as a nuisance variable in the later analyses. Specifically, we censored volumes with mean FD exceeding 1 mm and removed runs with remaining volumes less than 90% or mean FD greater than 0.5 mm. Additionally, we took participants’ in-scanner performance into account and excluded runs with low accuracy: 1) the average accuracy in any condition in each run less than 50% and 2) the average accuracy across conditions by run less than 66.7%. We included participants with at least three usable runs in the analyses, yielding a final sample of 33 children with four runs and 17 with three runs. In the final sample, the mean FD across participants was 0.23 ± 0.08 mm (range 0.1–0.42 mm), and there was a medium effect size of negative correlation between mean FD and age (*r*(48) = −0.24, *p* = 0.09; Cohen’s *d* = 0.5) (Fig. S1B).

### 2.8 Beta-series correlation analysis

We generated individual beta estimates for each trial following the estimation approach recommended by Mumford et al. (2012) to gain trial-specific activation estimates, similar to previous studies (e.g., Cisler et al., 2014; Ray et al., 2017; Turner et al., 2017). First, we used AFNI’s 3dDeconvolve to construct the design matrix. For a given condition of interest, we convolved that condition’s Guess period with a hemodynamic response function that modulated amplitude as a function of response time (‘dmBLOCK’ in AFNI), which is recommended when event durations vary based on response time (Grinband et al., 2008, Poldrack, 2015). In addition, the following nuisance regressors of no interest were also added into the design matrix: 1) the demeaned motion parameters and their derivatives; 2) volumes with FD greater than 1 mm; 3) the events of no interest including Guess period for all other conditions and Feedback period for all conditions. Thus, for each participant we constructed four design matrices, one for each condition of interest (i.e., Peer Mental, Peer Non-mental, Character Mental, and Character Non-mental). We then used AFNI’s 3dLSS to calculate the voxel-wise beta series of each trial using an iterative procedure to estimate task activity unique to each trial (Mumford et al., 2012). For example, for the Peer Mental condition with 24 trials, one general linear model (GLM) is conducted with a design matrix including two columns: one column for the first Peer Mental trial and the other column containing all other Peer Mental trials. Another GLM is then conducted with a design matrix including two columns: one column for the second Peer Mental trial and the other column containing all other Peer Mental trials. This iterative procedure is repeated until a beta estimate has been obtained for each individual trial (i.e., 24 separate GLMs). This procedure was then repeated similarly for Peer Non-Mental, Character Mental, and Character Non-Mental conditions.

**Fig. 1:**
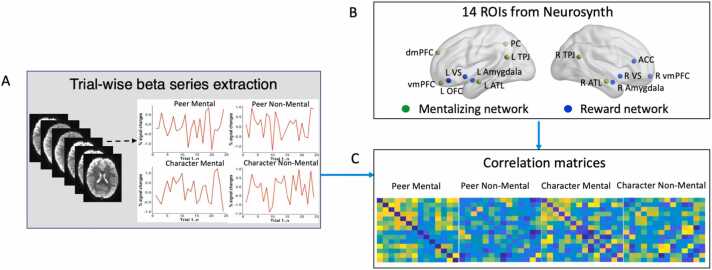
The fMRI data analysis procedure. (A) Trial-wise beta series were extracted from the Guess period for each condition and for each participant on the basis of separate general linear models. (B) Seven nodes for the mentalizing network were from Schmälzle et al. (2017) and seven nodes for the reward network were selected from meta-analysis in Neurosynth (http://neurosynth.org; Yarkoni et al., 2011). (C) Correlation matrices were calculated for each condition and for each participant by computing the correlation coefficients between beta-series of each ROI using Pearson’s correlation. Abbreviations: L, left; R, right; dmPFC, dorsomedial prefrontal cortex; vmPFC, ventromedial prefrontal cortex; TPJ, temporo-parietal junction; ATL, anterior temporal lobe; PC, precuneus; VS, ventral striatum; OFC, orbitofrontal cortex; ACC, anterior cingulate cortex.

The procedure of beta-series correlation analysis is shown in Fig. 1. Specifically, we selected 7 regions of interest (ROIs) for the mentalizing network, which were identical to those used in Schmälzle et al. (2017) and were derived from an automated meta-analysis of functional neuroimaging literature on the term “mentalizing” in Neurosynth (http://neurosynth.org; Yarkoni et al., 2011). Following the same procedure, we selected 7 ROIs for the reward network on the term “reward” in Neurosynth (http://neurosynth.org; Yarkoni et al., 2011). The details of all 14 ROIs are shown in Table 1 and visualized in Fig. 1B; all 14 ROIs were spatially non-overlapping. Finally, for each participant, we calculated correlation matrices for each condition based on the voxel-wise beta series by averaging beta series of each ROI sphere (5 mm radius) and computing the Pearson’s correlation between each ROI’s beta series, resulting in four correlation matrices for each participant (Fig. 1C).

**Table 1:** Regions of interest for the mentalizing network from Schmälzle et al. (2017) and for the reward network from Neurosynth (http://neurosynth.org).

Network	Region	MNI coordinates		
		x	y	z
**Mentalizing network** (Schmälzle at al, 2017)	Dorsomedial prefrontal cortex (dmPFC)	0	53	30
	Ventromedial prefrontal cortex (vmPFC)	0	48	-18
	Precuneus (PC)	0	-54	44
	Left temporo-parietal junction (L TPJ)	-48	-56	23
	Right temporo-parietal junction (R TPJ)	48	-56	23
	Left anterior temporal lobe (L ATL)	-53	-12	-16
	Right anterior temporal lobe (R ATL)	53	-12	-16
**Reward network**	Left orbitofrontal cortex (L OFC)	-22	36	-14
	Ventromedial prefrontal cortex (R vmPFC)	2	58	-8
	Anterior cingulate cortex (ACC)	2	32	16
	Left ventral striatum (L VS)	-12	10	-8
	Right ventral striatum (R VS)	12	10	-8
	Left amygdala	-20	-2	-14
	Right amygdala	24	-2	-18

### 2.9 Within- versus between-network connectivity

We first tested whether mentalizing and reward networks are functionally distinct by comparing within- versus between-network connectivity for each network using one-tailed paired *t*-tests. Specifically, we ran the tests on functional connectivity averaged across conditions. The results were corrected for multiple comparisons using the false discovery rate (FDR) correction (Benjamini and Hochberg, 1995) in R (“fdr” function).

### 2.10 Regression analyses on functional connectivity

In order to examine how social interaction modulates brain organization within and between the mentalizing and reward networks, we conducted regression analyses with linear mixed effects models on mean connectivity within and between these two networks using the ‘lme’ function in R (‘nlme’ package), separately. The within-network connectivity was calculated as the mean strength of all pairwise connectivity within the network (7 * 6/2 = 21 pairs of nodes for each network) and between-network connectivity as the mean strength of connectivity between nodes from different networks (7 * 7 = 49 pairs of nodes). In separate models for within mentalizing network, within reward network, and between mentalizing and reward networks, the mean connectivity was the dependent variable, and social interaction, mentalizing, and age as well as the interaction between social interaction and age were regressors of interest, with gender, mean FD, and IQ as regressors of no interest. Subjects were treated as a random effect in the model as we used a within-subject experimental design in the current study. For models showing significant main or interaction effects, post-hoc analyses were performed with linear mixed effects models. We determined that multicollinearity of the models was not a concern as no other independent variables except for the interaction term and its categorical variable (i.e., social interaction) had a variance inflation factor greater than 2 (Gareth et al., 2013).

Further, we examined how the involvement of brain regions within social-cognitive and social-motivational processing, including dmPFC, vmPFC, PC, bilateral TPJ, bilateral ATL, left OFC, right vmPFC, ACC, bilateral VS, and bilateral amygdala, is modulated by social interaction. Following previous studies (Costa et al., 2007; Gu et al., 2015), for each region, we computed its within-network strength (mean strength of functional connectivity between each region and other regions in the same network) and between-network strength (mean strength of functional connectivity between each region in one network and regions in the other network). The regression analyses of within-network and between-network strength of each region were implemented using similar linear mixed effects models as aforementioned, and the results were corrected for multiple comparisons using the FDR correction (Benjamini and Hochberg, 1995) in R (“fdr” function).

### 2.11 Brain–behavior correlation analyses

Next, we examined effects of social motivation and social interaction as well as their interaction on functional connectivity averaged across collapsed conditions of Peer and Character using linear mixed effects models. In separate models, social interaction (i.e., Peer, Character), behavior (i.e., RT or subjective reward), and their interaction were independent variables, mean functional connectivity (i.e., within mentalizing network, within reward network, and between mentalizing and reward networks) as a dependent variable, controlling for age, gender, mean FD, and IQ. Subsequently, for models showing significant main or interaction effects, post-hoc analyses were performed with linear mixed effects models. For subjective reward, we included children’s subjective reports of enjoyment, attention, and motivation, i.e., Liked Chatting, Liked Guessing, Felt When Matched, Paid Attention, and Wanted to See.

### 2.12 Specificity and control analyses

To verify the specificity of the effects shown in the mentalizing and reward networks, we ran a specificity analysis with two brain networks that are relevant to social interaction, i.e., mirror neuron and salience networks (for a review, see Redcay and Warnell, 2018), but these two networks were not predicted to be involved specifically in making predictions about a social partner within a text-based chat setting that did not include rejection. Further, we ran a control analysis with the motor network including bilateral somatomotor and paracentral regions which we did not predict to have any differential relation to the task conditions. All ROIs were selected from the meta-analyses of functional neuroimaging literature on terms “mirror”, “salience network”, and “motor network” separately and were identified with functional connectivity and coactivation maps in Neurosynth (http://neurosynth.org; Yarkoni et al., 2011), resulting in 10 ROIs for the mirror neuron network, 3 ROIs for the salience network, and 3 ROIs for the motor network (Figs. S3A–C**;** Table S2). All the ROIs were spatially non-overlapping. We performed regression analyses and brain–behavior correlation analyses within each of these networks using the same procedures as aforementioned.

## 3 Results

### 3.1 Behavioral results

**Fig. 2:**
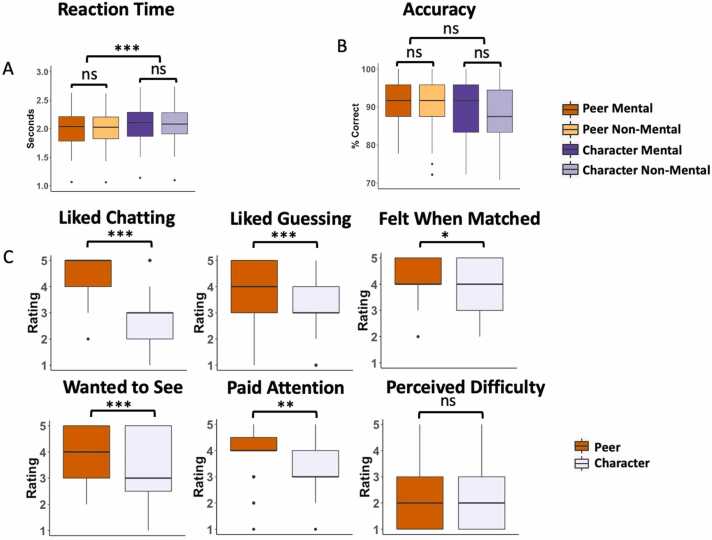
In-scanner performance and post-test assessments. (A) The regression analysis showed a main effect of social interaction on reaction time (*F*(1, 147) = 27.08, *p* < 0.001); participants responded more quickly to Peer versus Character trials (differences in averaged reaction time of collapsed conditions: *t*(49) = −4.7, *p* < 0.001). (B) For accuracy, there were no main effects of social interaction or mentalizing. (C) Children rated on a Likert-type scale of 1–5 which assessed their subjective reports of enjoyment, attention, motivation, and difficulty (i.e., Liked Chatting, Liked Guessing, Felt When Matched, Wanted to See, Paid Attention, and Perceived Difficulty); detailed questions for Peer and Character conditions are shown in Table S1. The Peer and Character conditions were compared by the Wilcoxon signed rank tests. * *p* < 0.05, ** *p* < 0.01, *** *p* < 0.001; ns, not significant.

Mean accuracy across all conditions was 90.17% (SD 7.49%) and mean RT was 2.04 s (SD 0.32). We examined the effects of social interaction, mentalizing, age, and the interaction between social interaction and age on accuracy and RT, with gender and IQ controlled in models. The results showed a main effect of social interaction on RT (*F*(1, 147) = 27.08, *p* < 0.001), and post-hoc pairwise *t*-test on mean RT of collapsed conditions revealed that children responded more quickly to Peer than Character condition (mean differences = −0.08 s, *t*(49) = −4.7, *p* < 0.001; Fig. 2A). There was also a main effect of age on RT (*F*(45) = 27.06, *p* < 0.001), with negative correlations between age and RT in both Peer and Character conditions (*ps* < 0.001). We did not observe a main effect of mentalizing or interaction effects of social interaction and age on RT. For accuracy, there was no main effect of social interaction but there was an interaction effect (Fig. 2B) of social interaction and age (*F*(1, 147) = 5.82, *p* = 0.02), which was driven by a positive relation between accuracy in the Character condition and age (*F*(45) = 5.46, *p* = 0.024) as shown in the post-hoc regression analysis controlling for gender and IQ.

We examined the effects of social interaction, age, and the interaction between social interaction and age on children’s subjective reports on the interactive task, controlling for gender and IQ. We found a main effect of social interaction on all examined reports (*p*s < 0.001) but Perceived Difficulty (*p* = 0.73). As the subjective reports were ordinal data (i.e., 5-point scale), we used paired Wilcoxon tests (i.e., Wilcoxon signed rank test) in post-hoc analysis. The follow-up Wilcoxon signed rank tests demonstrated that children reported more enjoyment, attention, and motivation during peer interaction compared to reasoning about the character (Fig. 2C and Table 2). The interaction effect of social interaction and age was seen in reports of Liked Chatting (*F*(1, 148) = 5.35, *p* = 0.022), Wanted to See (*F*(1, 148) = 6.48, *p* = 0.012), and Paid Attention (*F*(1, 148) = 16.3, *p* < 0.001) after correction for multiple comparisons using the FDR correction. However, in the post-hoc Spearman correlation analysis, these interaction effects did not show any significant effects between age and either Peer or Character condition; see Fig. S4.

**Table 2:** Subjective reports of experience assessed by the post-scan questionnaire.

Measure	Peer			Character			*p* (Wilcoxon signed rank test)
	median	mean±sd	range	median	mean±sd	range	
Liked Chatting	4.5	4.3 ± 0.81	2–5	3	2.76 ± 1.04	1–5	< 0.001
Liked Guessing	4	3.84 ± 0.96	1–5	3	3.26 ± 1.12	1–5	< 0.001
Felt When Matched	4	4.26 ± 0.83	2–5	4	3.96 ± 0.88	2–5	0.031
Wanted to See	4	4.08 ± 0.92	2–5	3	3.38 ± 1.23	1–5	< 0.001
Paid attention	4	3.9 ± 0.91	2–5	3	3.4 ± 0.97	1–5	0.002
Perceived difficulty	2	2.38 ± 1.23	1–5	2	2.2 ± 1.29	1–5	0.38

The relationships between RT and subjective reports of enjoyment, attention, and motivation when interacting with a peer were tested using the one-tailed Spearman’s correlation, and the results are shown in Table S3.

### 3.2 Greater within- versus between-network connectivity

For connectivity averaged across conditions, we observed significantly greater within-mentalizing versus between-network connectivity (*t*(49) = 9.96, *p* < 0.001; one-tailed paired *t*-test) and within-reward versus between-network connectivity (*t*(49) = 2.13, *p* = 0.02; one-tailed paired *t*-test) after correcting for multiple comparisons using the FDR correction.

### 3.3 Effects of social interaction on functional connectivity

**Fig. 3:**
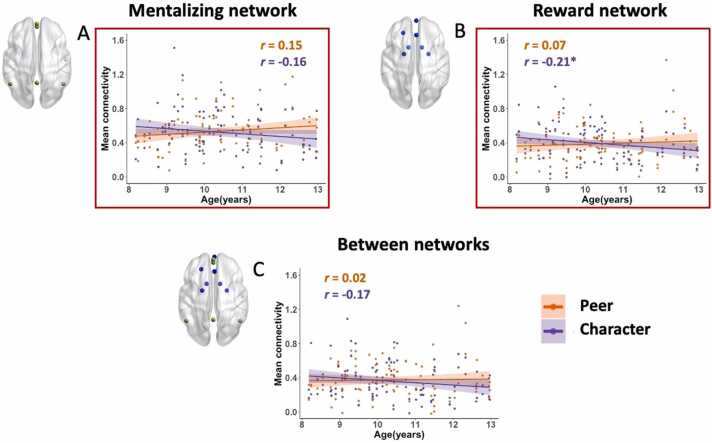
Results of regression analysis on mean functional connectivity within and between the mentalizing and reward networks. Scatterplots (A–C) depict interaction effects of social interaction and age. The significant interaction effects of social interaction and age (marked with red boxes) were seen within the mentalizing and reward network (mentalizing network: *F*(1, 147) = 9.4, *p* = 0.003; reward network: *F*(1, 147) = 7.13, *p* = 0.008); there was a marginal interaction effect of social interaction and age for between networks (*F*(1, 147) = 3.48, *p* = 0.064). Pearson’s correlation coefficients were calculated and are shown in the scatterplots. * *p* < 0.05. (For interpretation of the references to colour in this figure, the reader is referred to the web version of this article.)

In the mixed effects models, we examined the main effects of social interaction, mentalizing, age, and also the interaction effect of social interaction and age; in addition, gender, mean FD, and IQ were fixed effects of no interest, subjects as a random effect. Contrary to our hypotheses, there were no main effects of social interaction or mentalizing on functional connectivity within or between mentalizing and reward networks. There was also no main effect of age. However, there were interaction effects of social interaction and age on mean connectivity within the mentalizing and reward networks (mentalizing network: *F*(1, 147) = 9.4, *p* = 0.003; reward network: *F*(1, 147) = 7.13, *p* = 0.008) and a marginal interaction effect of social interaction and age for between networks (*F*(1, 147) = 3.48, *p* = 0.064); see Fig. 3. For the mentalizing and reward networks, the post-hoc regression tests did not show significant correlations between mean connectivity and age in the Peer (mentalizing network: *F*(45) = 1.41, *p* = 0.24; reward network: *F*(45) = 0.36, *p* = 0.55) or Character condition (mentalizing network: *F*(45) = 1.68, *p* = 0.2; reward network: *F*(45) = 3.25, *p* = 0.08).

In a follow-up exploratory analysis, we examined functional connectivity (averaged across collapsed conditions) between Peer versus Character condition in older children (upper quartile, i.e., age > 11.37 years) and younger children (lower quartile, i.e., age < 9.33 years) using two-tailed paired *t*-tests, separately. For older children, there was a pattern of greater connectivity in Peer relative to Character condition (mentalizing network: *t*(12) = 1.93, *p* = 0.078; reward network: *t*(12) = 0.93, *p* = 0.37; between network: *t*(12) = 0.68, *p* = 0.51); however, for younger children, the pattern is the opposite such that connectivity was greater in the Character compared to Peer condition (mentalizing network: *t*(12) = −2.33, *p* = 0.038; reward network: *t*(12) = −4.55, *p* = 0.001; between network: *t*(12) = −2.3, *p* = 0.04).

For the regression analysis on mentalizing and reward regions, we did not observe any significant results after correcting for multiple comparisons (See Supplementary Information–Results–Effects of social interaction on node connectivity strength).

### 3.4 Relations between functional connectivity and behavior

**Fig. 4:**
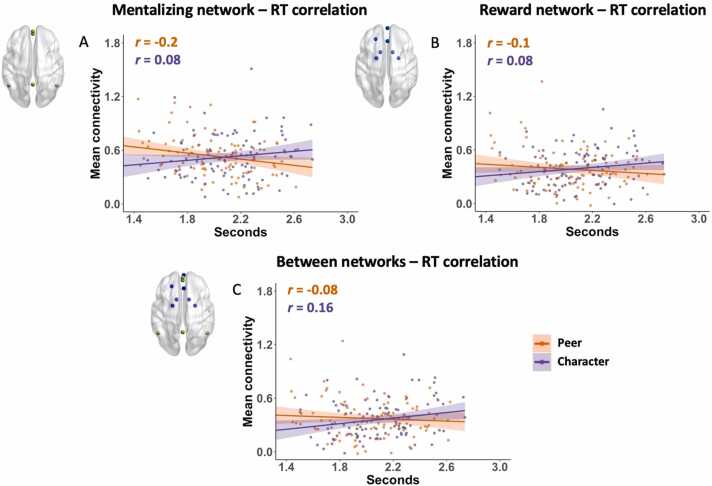
The relations between brain functional connectivity and reaction time (RT) in Peer and Character conditions. The scatterplots depict interaction effects of social interaction and RT on mean connectivity within and between the mentalizing and reward networks (mentalizing network: *F*(1, 146) = 13.77, *p* = 0.0003; reward network: *F*(1, 146) = 7.6*, p* = 0.007*;* between networks: *F*(1, 146) = 8.4, *p* = 0.004). Partial Pearson’s correlation coefficients controlling for age were calculated and are shown in the scatterplots.

We examined the main effects of social motivation (RT and subjective reward) and social interaction as well as the interaction effect of social motivation and interaction on functional connectivity within and between reward and mentalizing networks using mixed effects regression models. We did not find main effects of social interaction on within or between network connectivity (mentalizing network: *F*(1, 146) = 0.2, *p* = 0.65; reward network: *F*(1, 146) = 0.02*, p* = 0.89*;* between networks: *F*(1, 146) = 0.47, *p* = 0.49) or RT (mentalizing network: *F*(1, 146) = 0.52, *p* = 0.47; reward network: *F*(1, 146) = 0.00006*, p* = 0.99*;* between networks: *F*(1, 146) = 0.08, *p* = 0.78). However, we observed an interaction effect of social interaction and RT on mean connectivity within and between the mentalizing and reward networks (mentalizing network: *F*(1, 146) = 13.77, *p* = 0.0003; reward network: *F*(1, 146) = 7.6*, p* = 0.007*;* between networks: *F*(1, 146) = 8.32, *p* = 0.005). This interaction effect revealed that the brain–behavior relations in the Peer condition were significantly different from that in the Character condition (see Fig. 4A–C and Table 3), although functional connectivity averaged across Peer and Character conditions was not significantly correlated with mean RT (mentalizing network: *r*(48) = −0.02, *p* = 0.87; reward network: *r*(48) = 0.039, *p* = 0.79; between networks: *r*(48) = 0.12, *p* = 0.39). The post-hoc tests showed a pattern of positive relation between mean connectivity and RT in the Character condition (significant relation in between networks: *F*(48) = 4.37, *p* = 0.04) and a pattern of negative relations between mean connectivity and RT in the Peer condition (significant relation in the mentalizing network: *F*(48) = 4.31, *p* = 0.043).

**Table 3:** Brain–behavior correlation results. The interaction effects of social interaction and RT on connectivity within and between mentalizing and reward networks and post-hoc regression results.

Network	Interaction effect of social interaction * RT	Post-hoc regression analysis	
		Peer	Character
Mentalizing network	*F*(1, 146) = 13.72 ***	*F*(48) = 4.47 *	*F*(48) = 2.13
Reward network	*F*(1, 146) = 7.55 **	*F*(48) = 1.61	*F*(48) = 2.65
Between networks	*F*(1, 146) = 8.32 **	*F*(48) = 0.86	*F*(48) = 4.31 *

Note:* *p* < 0.05, ** *p* < 0.01, *** *p* < 0.001.

We did not see any main effects or interaction effects of social interaction and subjective reports of enjoyment, attention, and motivation on within- or between-network connectivity after correction for multiple comparisons.

### 3.5 Effects generalize to other social brain networks

The specificity analysis with the motor and the mirror neuron networks did not show any main or interaction effects on within-network connectivity. However, we found an interaction effect of social interaction and age on mean connectivity within the salience network (*F*(1, 147) = 5.87, *p* = 0.017); post-hoc regression tests showed a significant positive association between age and connectivity in the Peer condition (*F*(45) = 5.68, *p* = 0.02) but not in the Character condition (*F*(45) = 0.76, *p* = 0.39). Thus, the interaction effects of age and social interaction on mean connectivity reported above are not specific to reward and mentalizing networks alone, but extend to other networks associated with social interaction. In addition, we tested the effect of the brain–behavior relations in RT and subjective reports of reward with motor regions, mirror neuron, and salience networks. The results showed an interaction effect of RT and social interaction in the mean connectivity within the mirror neuron network (*F*(1, 147) = 7.49, *p* = 0.007), and post-hoc regression tests demonstrated negative correlations between mean connectivity and RT in the Peer condition (*F*(48) = 4.36, *p* = 0.04), although there was no correlation between connectivity averaged across all conditions and RT (*r*(48) = −0.12, *p* = 0.4). No other main or interaction effects were found in these control regions or networks.

### 3.6 Validation of results with low age-motion correlation sample

Given the medium effect size of correlation between age and head motion (*r* = −0.24, Cohen’s *d* = 0.5), to test whether observed findings were driven by the head motion, we ran the aforementioned analyses in a subsample (n = 30) where motion and age were uncorrelated. We observed similar results in this low age-motion correlation group (for details see Supplementary Information–Results–Validation of results with low age-motion correlation sample), indicating the results in the full sample were not driven by head motion.

## 4 Discussion

The current study brings a social-interactive neuroscience approach (Redcay and Schilbach, 2019, Redcay and Warnell, 2018, Schilbach et al., 2013) and a brain network perspective to examine the neural mechanisms underlying social interaction in middle childhood. Contrary to our hypotheses, neither social interaction nor mentalizing demonstrated main effects on functional connectivity. Instead, the effect of social interaction on functional connectivity was modulated by both age and behavioral performance. Specifically, as age increased the differences in mean functional connectivity between Peer and Character conditions got larger within and between mentalizing and reward networks. This age-dependent effect was also seen in the salience network, but not other networks (i.e., motor and mirror neuron networks). Further, brain functional connectivity between the mentalizing and reward networks associated with children’s reaction time to peer, reflecting a link between stronger brain coupling during social interaction and faster responses to the Peer condition. This effect of RT was also present in the mirror neuron network, but not the other control networks (i.e., motor and salience networks). Collectively, these findings suggest that age-related increases in functional connectivity among brain regions support social processing—beyond the mentalizing and reward networks—and that this increasing connectivity may relate to increasing motivation (e.g., faster response) to engage with a social partner from middle childhood to adolescence.

### 4.1 Age-related differences in network connectivity during social interaction

Previous studies have highlighted involvement of both the mentalizing and reward networks in social interaction (Alkire et al., 2018, Chevallier et al., 2012, Redcay et al., 2010, Rilling and Sanfey, 2011). Theoretical work suggests social interaction may lead to greater functional connectivity among networks relevant to social processing (Redcay and Schilbach, 2019, Ruff and Fehr, 2014, Schilbach et al., 2013). Further, coactivations of mentalizing and reward regions are related to social reward processing (e.g., Izuma et al., 2008; Powers et al., 2013). However, in the current study the hypothesis that making predictions about a peer would lead to greater connectivity within and between these networks than making predictions about a story character was not supported, as there was no significant main effect of social interaction.

One possible account for the lack of an effect of social interaction is that our hypotheses were based on the adult literature, and thus these effects may be emerging during childhood into adolescence (Devine and Hughes, 2013, Farmer et al., 2015, Nelson et al., 2016, Parker et al., 2015). In support of this, we found that effects of social interaction on brain connectivity were modulated by age. The increasing connectivity within the mentalizing network during this period is consistent with previous studies showing age-related changes in functional connectivity within the mentalizing network during mental state reasoning (Gweon et al., 2012, Richardson et al., 2018, Mukerji et al., 2019). Our data also accord with findings that social context, including social rewards and peer evaluation, can alter neural activity within the reward and mentalizing networks, and that age modulates these effects (Guyer et al., 2012, Somerville et al., 2013, Warnell et al., 2018).

Notably, our findings differ from those reported in univariate activation analyses using the same task (Alkire et al., 2018). Specifically, our previous study found decreasing activation for Peer versus Character condition with age within social-cognitive regions, whereas here we found increasing functional connectivity for Peer versus Character condition with age within similar regions. One account for this discrepancy is that large activation may “quench” neural variability between regions, which can lead to decreased functional connectivity (Cole et al., 2021, Ito et al., 2020). That is, the younger children show greater activation for Peer versus Character condition, which perhaps reduces the regional variability, leading to less functional connectivity. Since the difference in activation between Peer versus Character reduces with age, we are then able to observe the functional connectivity differences. Nevertheless, future studies are needed to further elucidate the potential reasons for the differences in activation versus functional connectivity findings.

### 4.2 The linkage between functional connectivity and individual differences in behavior

Functional connectivity during social interaction covaried with participants’ reaction time to Peer conditions, and this brain–behavior relation was significantly different between Peer and Character conditions, suggesting these connectivity patterns contribute to social-interactive performance. Overall, children who responded faster when interacting with a social partner showed greater functional connectivity within and between mentalizing and reward networks. The faster response to a peer may be attributed to heightened motivation when interacting with a social partner compared to guessing about a character. Expecting a social reward (i.e., positive feedback from a peer) may lead to greater connectivity within the reward network, resulting in the relation between faster response and greater connectivity within the reward network during social interaction. Similarly, greater functional connectivity within the mentalizing network and between mentalizing and reward networks may relate to the motivation to “succeed” in a social interaction (e.g., making a correct guess about a social partner, receiving a matched response from a peer). As such, this linkage between greater functional connectivity within and between mentalizing and reward networks and faster reaction time is consistent with theoretical and empirical work suggesting that engaging with a social partner is motivating (Chevallier et al., 2012, Pfeiffer et al., 2014, Schilbach et al., 2010). However, the brain–behavior relation observed here may be driven by the negative correlation pattern between functional connectivity and RT in the Character condition. This finding is not expected; because the Character condition would not elicit reward engagement, we would not expect a systematic relation between reward functional connectivity and RT in Character condition. This somewhat surprising finding needs further research to elucidate.

Participants also reported greater enjoyment of and motivation for interacting with the peer than reasoning about the story character. However, despite our predictions, these self-reports did not relate to connectivity within or between networks during social interaction, nor was this relation different from the relation between participant’s reported enjoyment and motivation for the story character and connectivity during the Character condition. The lack of a relation might be due to little variability in the self-report measures of subjective reward in the Peer condition, which limits the power to detect their potential relations with brain connectivity. For example, 82% of children rated a 4 or 5 out of 5 for their enjoyment of chatting with the peer, and 80% of children rated a 4 or 5 out of 5 for their enjoyment when their answer matched the answer from the peer. However, the subjective report of enjoyment for answering questions about the character showed decent variability (36% rated 1 or 2; 48% rated 3; 16% rated 4 or 5). Thus, it is likely to reflect high enjoyment chatting with peer instead of minimal variability in enjoyment across conditions. Nevertheless, future studies could shed light on this point by designing measures of subjective reward that elicit better variability, especially in social interaction.

### 4.3 Mirror neuron and salience networks demonstrate behavior-relevant functional connectivity during social interaction

Our a priori hypotheses were that the mentalizing and reward networks would show greater connectivity during this task in which participants made predictions about a social partner compared to a character. While salience and mirror neuron networks are also associated with social interaction, the text-based contexts in the current study were not a priori expected to engage them. Here, though, we did find that, like the mentalizing and reward networks, the salience network demonstrated greater connectivity during social interaction with greater age. The salience network has been found to be activated when directing attention to self-relevant and salient events, such as physical and emotional pain (for a review, see Menon and Uddin, 2010). Although the involvement of the salience network during social interaction is mostly documented in studies of social rejection (for a review, see Wang et al., 2017), it has also been shown that regions of this network are sensitive to social interaction, especially when reciprocal and contingent responses from a human partner are present (Guionnet et al., 2012, Redcay et al., 2010). Recent studies have shown an increase of involvement of the salience networks with age in non-interactive contexts (e.g., Biagi et al., 2016; Jones et al., 2014; Shaw et al., 2012). Our findings provide primary evidence that the salience network is functionally connected more in social-interactive versus non-interactive contexts as children get older, suggesting that connectivity during social interaction may be a more general feature of social brain networks as children enter adolescence.

While the mirror neuron network did not show age-related functional connectivity changes during social interaction, it did demonstrate a relation to behavior, specifically reaction time. The mirror neuron network encompasses regions associated with goal-directed action in adults (Biagi et al., 2016, Morales et al., 2019) and children (Biagi et al., 2016, Morales et al., 2019, Reynolds et al., 2019, Reynolds et al., 2015), which may be related to its role in responding via button press. Additionally, prior work has shown that the mirror neuron network is modulated by social context. For example, it is engaged when processing communicative intent and demonstrates greater functional connectivity with the mentalizing network in communicative versus non-communicative contexts (Ciaramidaro et al., 2014, Ciaramidaro et al., 2007, Schippers et al., 2010, Schippers et al., 2009, Sperduti et al., 2014). Thus, one account for the greater connectivity within the mirror neuron network may be related to the role of this network in recognizing communicative intent when making predictions about a social partner versus a story character.

### 4.4 Limitations

A few limitations are worth noting when interpreting these findings. First, this fMRI task was a highly-structured, chat-based interaction that was built on an illusion of a live, real-time social partner (Alkire et al., 2018, Warnell et al., 2018). Though we have taken extensive precautions to ensure participants believed the illusion—including the post-scan questionnaire and debriefing—this experience still differs from a real-world social interaction. In real life, social interactions contain many components, such as gestures, eye gaze, facial expressions, tones, and physical features, and all of these components can significantly affect cognitive and emotional processing. Future attempts to develop more ecologically valid social interaction tasks during fMRI data acquisition would provide greater insight into the neural underpinnings of real-world social interactions.

Second, the relatively small number of trials may affect the reliability of the beta series functional connectivity analysis. In the current study, there were only 24 trials for each condition. Research has shown that more trials tend to have greater power for beta series functional connectivity (Cisler et al., 2014). Nevertheless, as shown in Cisler et al. (2014), beta series functional connectivity analysis with 24 trials would still outperform other functional connectivity analysis (i.e., psychophysiological interaction analysis).

Third, we only explored the brain–behavior relations with participants’ in-scanner performance and self-reported subjective reward assessed by the post-scan questionnaire. However, the behavioral relevance of brain organization during social interaction can be further explored by examining correlations with measures of real-world social networks (Schmälzle et al., 2017), or subjective experience of social rewards in real life.

## 5 Conclusions

In sum, our data provide evidence for the development of brain mechanisms supporting social interaction in the context of a real-time interaction. We found that functional connectivity within mentalizing, reward, and salience networks during social interaction changes with age over middle childhood. The brain–behavior relations demonstrated that responding faster to the Peer than Character condition was related to greater connectivity within and between mentalizing and reward networks, as well as within the mirror neuron network. These results highlight the increasing importance of the mentalizing, reward, mirror neuron, and salience networks, as well as the interactions between mentalizing and reward networks in responding to social interaction, as children get older. The findings demonstrate how functional connectivity analysis may provide complementary information to univariate activation results (Alkire et al., 2018) and facilitate a more comprehensive understanding of brain function during social interaction. Furthermore, these findings from typically developing children may serve as a baseline for investigating atypical brain organization underlying social difficulties in developmental disorders, such as autism spectrum disorder.

## Data statement

The tidy data used in the current study are publicly available at https://github.com/Yaqiongxiao/SocialfcMRI.

## Declaration of Competing Interest

The authors declare no competing interests.

## Data Availability

The tidy data used in this study and completed R code for implementing all the analyses are publicly available at https://github.com/Yaqiongxiao/SocialfcMRI.
