# Supplementary material for: Developmental differences in brain functional connectivity during social interaction in middle childhood
Source: Dev Cogn Neurosci. 2022 Jan 31;54:101079. doi: 10.1016/j.dcn.2022.101079 (PMC9019834; doi:10.1016/j.dcn.2022.101079)
Supplement: Supplementary file 1 — Supplementary material [file mmc1.docx]

**Supplementary Information**

**Results**

**Effects of social interaction on node connectivity strength**

We then examined the interaction of social interaction and age on the within-network and between-network strength of mentalizing and reward regions. Interaction effects were found in dmPFC (*F*(1, 147) = 4.91, *p* = 0.028) for within-mentalizing network strength and in ACC (*F*(1, 147) = 8, *p* = 0.005) for within-reward network strength. For between-network strength, interaction effects were found in dmPFC (*F*(1, 147) = 5.8, *p* = 0.017), left TPJ (*F*(1, 147) = 5.17, *p* = 0.024), left ATL (*F*(1, 147) = 5.5, *p* = 0.02), ACC (*F*(1, 147) = 5.82, *p* = 0.017), and left VS (*F*(1, 147) = 4.79, *p* = 0.03). The multiple comparisons were corrected for a total of 28 interactions (14 for within-network strength of mentalizing and reward regions and 14 for between-network strength of mentalizing and reward regions) using the FDR correction with an adjusted *p* value < 0.05 ('fdr’ method in R; Benjamini and Hochberg, 1995).

**Validation of results with low age-motion correlation sample**

To test whether the results were driven by head motion, we first randomly sampled sets of 30 participants and selected the group with a low correlation (*r*(28) = -0.07, Cohen’s *d* = 0.15) and then ran the analyses with this subset of data.

(1) Within- versus between-network connectivity

There were significant differences between within-mentalizing versus between-network connectivity (*t*(29) = 8.02, *p* < 0.001) and within-reward versus between-network connectivity (*t*(29) = 2.14, *p* = 0.02).

(2) Effects of social interaction on functional connectivity

There were no main effects of social interaction or mentalizing or age. We observed interaction effects of social interaction and age on mean connectivity (mentalizing network: *F*(1, 87) = 7.12, *p* = 0.009; reward network: *F*(1, 87) = 6.04, *p* = 0.016; between networks: *F*(1, 87) = 4.06, *p* = 0.047). For older children, there were no significant differences between Peer and Character conditions (mentalizing network: *t*(8) = 1.95, *p* = 0.19; reward network: *t*(8) = 0.91, *p* = 0.46; between network: *t(*8) = 1.3, *p* = 0.34). For younger children, there was significant difference or a trend of differences between Peer and Character conditions (mentalizing network: *t*(8) = -1.93, *p* = 0.07; reward network: *t*(8) = -2.17, *p* = 0.044; between network: *t*(8) = -1.13, *p* = 0.27).

(3) Relations between functional connectivity and behavior

There were no main effects of social interaction or reaction time (RT) on functional connectivity. We observed significant interaction effects of social interaction and RT on mean connectivity (mentalizing network: *F*(1, 86) =9.07, *p* = 0.003; reward network: *F*(1, 86) = 4.35, *p* = 0.04; between networks: *F*(1, 86) = 5.9, *p* = 0.02). We did not observe any main effects or interaction effects of social interaction and subjective reports of enjoyment, attention, and motivation on within- or between-network connectivity after correcting for multiple comparisons.

(4) Effects generalize to other social brain networks

We found an interaction effect of social interaction and age on mean connectivity within the mirror neuron network (*F*(1, 87) = 4.5, *p* = 0.036). For the brain—behavior correlation, there was an interaction effect of RT and social interaction in the mean connectivity within the mirror neuron network (*F*(1, 86) = 8.36, *p* = 0.005).

**Correlations between the salience network and both mentalizing and reward networks**

We examined the relationships between the salience network and both mentalizing and reward networks with mean connectivity of all four conditions using paired *t*-tests. There were significant correlations between the salience network and the mentalizing network (*r*(48) = 0.47, *p* < 0.001) and between the salience network and the reward network (*r*(48) = 0.53, *p* < 0.001), suggesting functional connectivity within the salience network is highly correlated with functional connectivity within mentalizing and reward networks.

**Correlations between the mirror neuron and mentalizing networks**

We examined the relationships between mean functional connectivity—averaged across conditions of Peer and Character separately—in the mirror neuron and mentalizing networks. There were significant correlations between the mirror neuron and mentalizing networks in both Peer (*r*(98) = 0.68, *p* < 0.001) and Character (*r*(98) = 0.64, *p* < 0.001) conditions, which is inconsistent with previous findings showing greater functional connectivity between the mirror neuron and mentalizing networks in communicative versus non-communicative contexts (Ciaramidaro et al., 2014; Schippers et al., 2010; Sperduti et al., 2014). We infer that it might be due to the role of the mirror neuron network in goal-directed action (e.g., responding via button press) in both Peer and Character conditions.

**Table S1. Post-scanner questions about the subjective experience on the interactive task**

| Item | Peer | Character |
| --- | --- | --- |
| Liked Chatting | How much did you like chatting with [partner name]? Where 1 is not at all and 5 is a lot. | How much did you like it when you were just answering the computer? Where 1 is not at all and 5 is a lot. |
| Liked Guessing | How much did you like guessing what s/he would pick based on his/her hints? Where 1 is not at all and 5 is a lot. | How much did you like guessing what would come next in the story based on the hints? Where 1 is not at all and 5 is a lot. |
| Felt When Matched | How did you feel when she/he agreed with your answer? Where 1 is very bad and 5 is very good. | How did you feel when your answer matched the computer answer? Where 1 is very bad and 5 is very good. |
| Wanted to See | How much did you want to see if s/he picked the same as you? Where 1 is not at all and 5 is a lot. | How much did you want to see if the computer answer matched your answer? Where 1 is not at all and 5 is a lot. |
| Paid Attention | Sometimes it can be hard to pay attention when you’re playing games. How much did you pay really, really close attention answering a question when he/she was the one you were chatting with? Where 1 is not at all and 5 is a lot. | Sometimes it can be hard to pay attention when you’re playing games. How much did you pay really, really close attention answering a question when the computer was giving the hints? Where 1 is not at all and 5 is a lot. |
| Perceived Difficulty | How hard was it to guess what [partner name] would pick? Where 1 is very easy and 5 is very hard. | How hard was it to guess what would come next in the story? Where 1 is very easy and 5 is very hard. |

**Table S2. Regions of interest for mirror neuron, salience, and motor networks from the meta-analyses in Neurosynth (http://neurosynth.org)**

| **Network** | **Region** | **MNI coordinates** | | |
| --- | --- | --- | --- | --- |
|  |  | x | y | z |
| **Mirror neuron**  **network** | Left superior parietal lobe | -24 | -56 | 66 |
|  | Right superior parietal lobe | 24 | -58 | 66 |
|  | Left inferior parietal lobe | -34 | -46 | 56 |
|  | Right inferior parietal lobe | 34 | -46 | 54 |
|  | Left middle temporal gyrus | -46 | -66 | 4 |
|  | Right middle temporal gyrus | 50 | -62 | 2 |
|  | Left dorsal premotor | -34 | -28 | 56 |
|  | Right dorsal premotor | 34 | -20 | 54 |
|  | Left inferior frontal gyrus | -58 | 8 | 26 |
|  | Right inferior frontal gyrus | 58 | 10 | 20 |
| **Salience network** | Dorsal anterior cingulate cortex | 4 | 32 | 20 |
|  | Left anterior insula | -38 | 14 | -6 |
|  | Right anterior insula | 36 | 14 | 6 |
| **Motor network** | Left somatomotor cortex | -39 | -18 | 51 |
|  | Right somatomotor cortex | 41 | -13 | 53 |
|  | Paracentral lobule | -3 | 1 | 49 |

**Table S3. Relationships between reaction time (RT) and subjective reports of enjoyment, attention, and motivation when interacting with a peer**

|  | *rho* | *p* |
| --- | --- | --- |
| Liked Chatting | -0.12 | 0.2 |
| Liked Guessing | -0.07 | 0.33 |
| Felt When Matched | -0.25 | 0.038 |
| Paid Attention | -0.03 | 0.42 |
| Wanted to See | -0.24 | 0.048 |


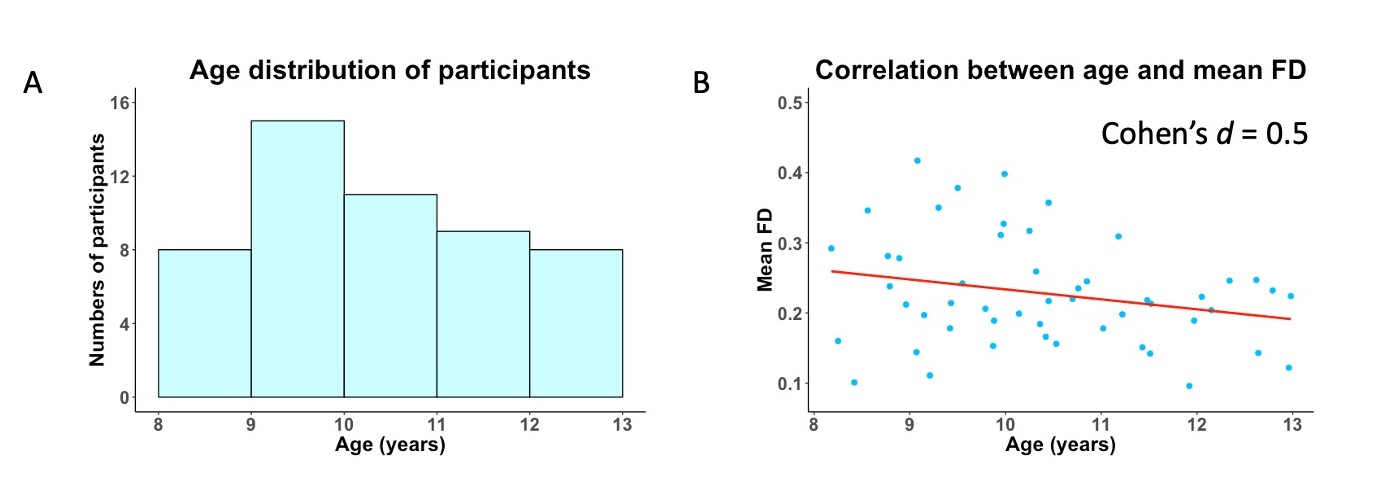


Fig. S1. Age distribution of participants in the present study (A) and the relations between age and head motion (mean framewise displacement, mean FD) (B). The scatterplot indicates a medium effect of negative correlation between age and mean FD (*r*(48) = -0.24, *p* = 0.09; Cohen’s *d* = 0.5).


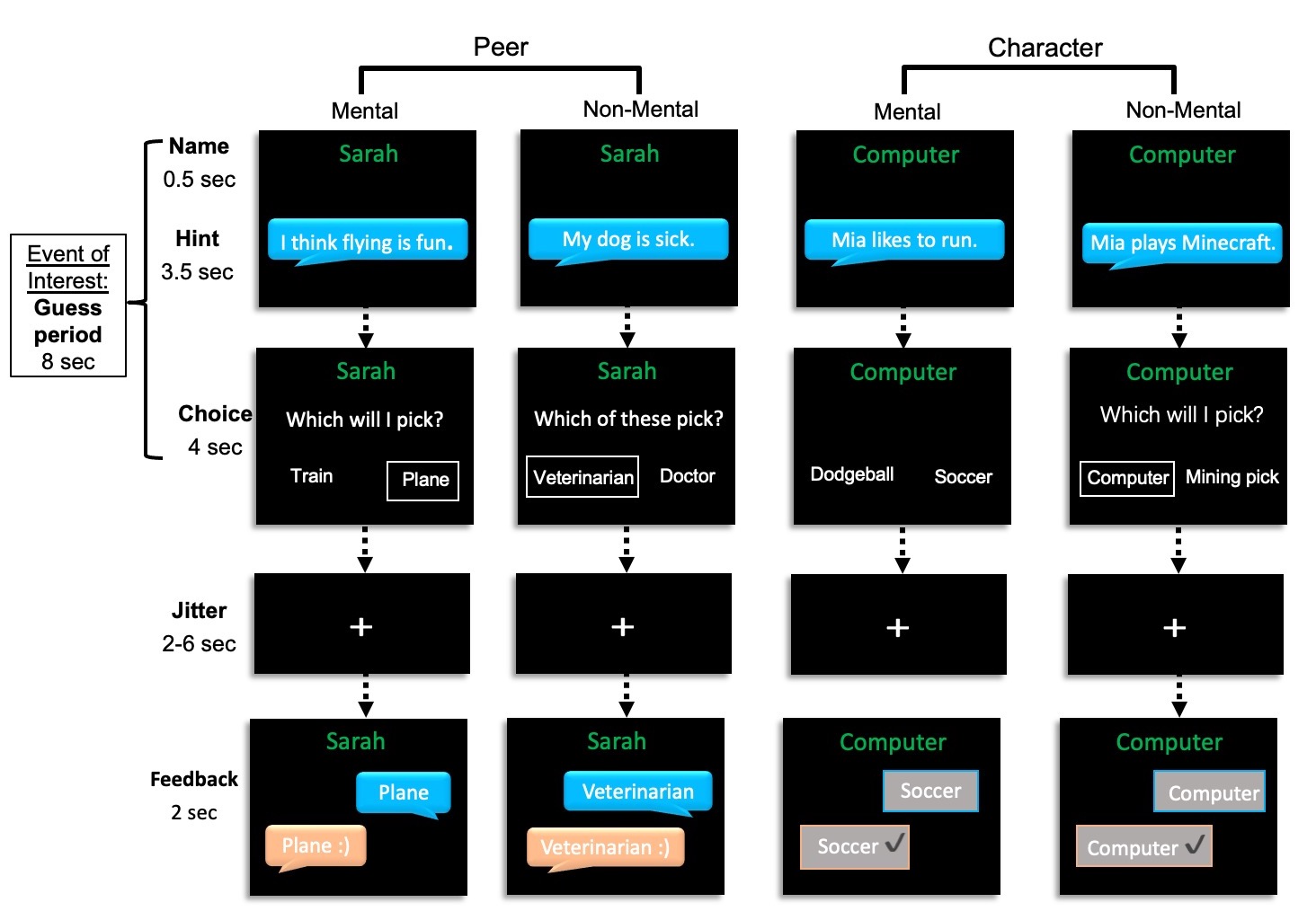


Fig. S2. The fMRI task. There are four conditions in this 2 * 2 event-related design, i.e., Peer Mental, Peer Non-Mental, Character Mental, and Character Non-Mental, with 24 trials for each condition. In Peer conditions, children believed they were chatting with a real person, while in Character conditions, children knew they were receiving responses from the computer. The Mental conditions required mental state reasoning (e.g., thinking, desire, belief, or emotion), whereas the Non-Mental conditions did not require mental state reasoning. Each trial was comprised of two periods: Guess (8 s) and Feedback (2 s). If the participant’s answer matched that from the peer or the computer, either a smiley face (Peer) or a check mark (Character) was shown in the Feedback period.


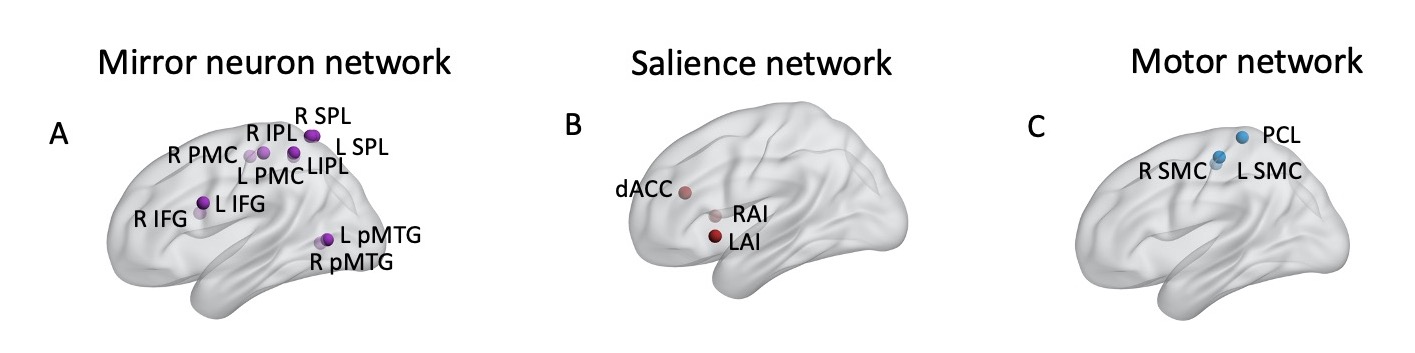


Fig. S3. Regions of interest in control networks: (A) mirror neuron network, (B) salience network, and (C) motor network. L, left; R, right; PMC, premotor cortex; IFG, inferior frontal gyrus; IPL, inferior parietal lobe; SPL, superior parietal lobe; dACC, dorsal anterior cingulate cortex; AI, anterior insula; SMC: somatomotor cortex; PCL: paracentral lobule.


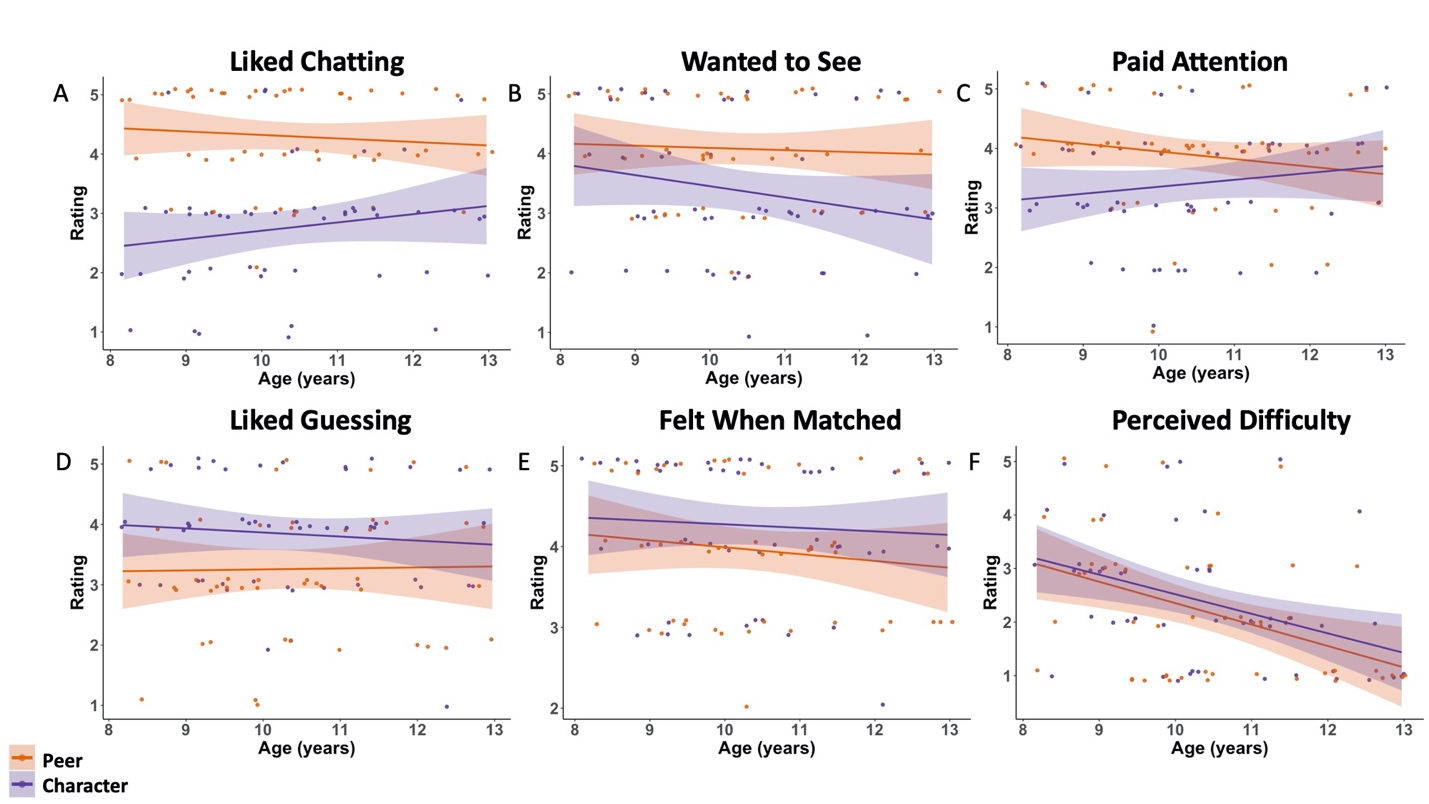


Fig. S4. Significant interaction effects of social interaction and age in reports of: (A) Liked Chatting (*F*(1, 148) = 5.35, *p* = 0.022), (B) Wanted to See (*F*(1, 148) = 6.48, *p* = 0.012), and (C) Paid Attention (*F*(1, 148) = 16.3, *p* < 0.001), but not in reports of: (D) Liked Guessing (*F*(1, 148) = 1.69, *p* = 0.2), (E) Felt When Matched (*F*(1, 148) = 0.51, *p* = 0.49), and (F) Perceived Difficulty (*F*(1, 148) = 0.21, *p* = 0.65). For reports with significant interaction effects of social interaction and age, the post-hoc analysis did not show any significant effects in either Peer or Character condition.

**References**

Benjamini, Y., Hochberg, Y., 1995. Controlling the False Discovery Rate: A Practical and Powerful Approach to Multiple Testing. Journal of the Royal Statistical Society. Series B (Methodological) 57, 289–300.

Ciaramidaro, A., Becchio, C., Colle, L., Bara, B.G., Walter, H., 2014. Do you mean me? Communicative intentions recruit the mirror and the mentalizing system. Soc Cogn Affect Neurosci 9, 909–916. https://doi.org/10.1093/scan/nst062

Schippers, M.B., Roebroeck, A., Renken, R., Nanetti, L., Keysers, C., 2010. Mapping the information flow from one brain to another during gestural communication. PNAS 107, 9388–9393. https://doi.org/10.1073/pnas.1001791107

Sperduti, M., Guionnet, S., Fossati, P., Nadel, J., 2014. Mirror Neuron System and Mentalizing System connect during online social interaction. Cogn Process 15, 307–316. https://doi.org/10.1007/s10339-014-0600-x
